# Socioeconomic inequalities in primary care research: how can we make research more equitable? A quantitative and qualitative service evaluation

**DOI:** 10.3399/BJGPO.2025.0082

**Published:** 2026-05-20

**Authors:** Alison Janes, Helen Riding, Shona Haining, Morag Burton, Hilary Allan, Sarah Sowden

**Affiliations:** 1 NHS North of England Commissioning Support Unit (NECS), Newcastle Upon Tyne, UK; 2 Population Health Sciences Institute, Newcastle University, Newcastle upon Tyne, UK; 3 NHS North East and North Cumbria Integrated Care Board, Newcastle upon Tyne, UK; 4 National Institute for Health and Care Research (NIHR) Applied Research Collaboration (ARC) for the North-East and North Cumbria (NENC), Newcastle upon Tyne, UK; 5 National Institute for Health and Care Research (NIHR) Regional Research Delivery Network (RDN) for the North-East and North Cumbria (NENC), Newcastle upon Tyne, UK

**Keywords:** inequalities, research methods, qualitative research, research, health inequities

## Abstract

**Background:**

Health research should reflect contexts where interventions are needed to provide evidence generalisable to practice. There is concern that socioeconomically disadvantaged populations with poorer health and care outcomes are underrepresented in research, potentially exacerbating health inequalities. Evidence on the relationship between socioeconomic deprivation and general practice research involvement is limited.

**Aim:**

To explore the relationship between socioeconomic deprivation, general practice research involvement, and recruitment.

**Design & setting:**

Quantitative analysis of routine data and qualitative interviews with clinical and non-clinical general practice staff in the North East and North Cumbria (NENC) area of England.

**Method:**

Analysis of general practice and National Institute for Health and Care Research (NIHR) Clinical Research Network (now the Regional Research Delivery Network [RDN]) data to understand associations between neighbourhood deprivation and research recruitment. Semi-structured interviews with GPs and practice managers or administrators explored barriers and facilitators to research involvement and patient recruitment in disadvantaged areas. Interviews were recorded, transcribed, and thematically analysed.

**Results:**

Research recruitment was lower among practices (*n* = 358) with more deprived populations (χ^2^(4) = 7520.8, *P*<0.001), and significantly lower among ‘Deep End’ practices in areas of blanket socioeconomic disadvantage (*P*<0.001). Eleven GPs and four practice managers or administrators were interviewed. Barriers included greater difficulty recruiting patients, resource pressures, limited financial benefit, and awareness. Supporting factors were research designed to meet needs of socioeconomically disadvantaged populations that was accurately costed, remunerated, and resourced, with concise, practically focused communications.

**Conclusion:**

To support equitable general practice research participation, research should be relevant for and designed to meet the needs of socioeconomically disadvantaged populations, and be accurately costed and remunerated. Targeting additional resource at practices in socioeconomically disadvantaged areas could incentivise equitable research participation.

## How this fits in

Any systematic underrepresentation of socioeconomically disadvantaged populations in health research limits the evidence base for appropriate and effective interventions, and risks exacerbating health inequalities. This evaluation found lower levels of research recruitment in general practices where more patients live in the most deprived neighbourhoods (χ^2^(4) = 7520.8, *P*<0.001). Barriers to practice research involvement in areas of blanket deprivation included a high volume of complex patient need; more time-consuming patient recruitment; limited resources; limited financial benefits; and limited awareness of research opportunities. For benefits of primary care research participation to be distributed more equitably, research should be designed to meet needs of socioeconomically disadvantaged patients, and be accurately costed, remunerated, and resourced to reflect additional effort involved.

## Introduction

There is concern underrepresentation of socioeconomically disadvantaged populations in health research may result in limited evidence of appropriate and effective interventions for these populations,^1^ exacerbating health inequalities. Socioeconomic disadvantage is a key determinant of health, associated with higher prevalence of disease.^2,3^ A study of geographical inequalities in recruitment to health research in England found patient recruitment is disproportionately lower in areas with higher prevalence of long-term and mental health conditions.^4^

Inequalities in research recruitment may widen health inequalities, as research often focuses on healthier populations, limiting generalisability to communities facing greater health challenges owing to differences in population risk factors, outcomes, and contexts.^2,4,5^ This means clinicians serving populations with the highest level of healthcare need are less able to access evidence of appropriate and effective interventions for these populations. Measurement of inequalities in research recruitment is challenging as socioeconomic status of participants is often not gathered or reported.^1^

Guidance in England^6,7^ and the USA^8^ aims to make health research more inclusive and representative of the communities it is meant to serve. Primary care has been described as a ‘*sleeping giant*’ in its potential to contribute to research delivery, increase diversity of clinical trial populations, and generate evidence for patient care.^9^ To our knowledge there is no evidence of the scale to which lack of research inclusivity manifests in general practice.

Aligning the geographic footprint of each practice with neighbourhood deprivation data has enabled measurement of GP practice socioeconomic deprivation and development of ‘Deep End’ Networks. These are groups of practices serving areas of blanket socioeconomic deprivation aiming to reduce health inequalities and address the ‘inverse care law’,^10^ the principle that those most in need often have the least access to healthcare resources.^11^ There is limited published evidence to understand what supports these practices and their practice populations to engage with research. Drawing on understanding of inequalities in access, use, experience, and outcomes of health care more broadly, this is likely to reflect a complex interaction of patient, provider, and wider system factors.^12–14^ This service evaluation explored the relationship between socioeconomic deprivation, general practice research recruitment, and perspectives on research involvement.

## Method

### Design

This was a mixed-methods service evaluation, carried out to inform quality improvement of National Institute for Health and Care Research (NIHR) Clinical Research Network (CRN) services in the North East and North Cumbria (NENC) area. The NIHR CRN is now known as the Regional Research Delivery Network (RDN), and to minimise confusion this terminology will be used henceforth. Quantitative analysis was carried out to understand the relationship between practice deprivation and research recruitment; this informed semi-structured qualitative interviews with GPs and practice managers to explore barriers and facilitators to research involvement and patient recruitment in socioeconomically disadvantaged areas.

### Setting

NENC is the largest NHS integrated care board (ICB) geography in England, covering a population of approximately 3.2 million. This includes the urban centres of Tyne and Wear and Teesside, containing local authorities with some of the highest levels of blanket deprivation in England.^15^ NENC is one of 15 sub-regions in England with a local service to support research delivery funded by the NIHR as the RDN. NENC is a mid-sized region in terms of overall research participant recruitment. Primary care participant recruitment is relatively low; each year 30–40% of GP practices in the region are involved in recruitment of research participants on site and/or as a Participant Identification Centre (PIC).

### Measures

GP practice research recruitment was measured using data from the NIHR RDN Open Data Platform (ODP); at the time, this was known as the NIHR CRN ODP.^16^ This reports aggregate number of patients recruited to NIHR RDN portfolio research studies for each general practice in NENC by financial year. The NIHR RDN portfolio consists of health research studies that apply for support from the NIHR RDN in England and meet eligibility criteria: research studies with appropriate ethical approval and full research cost funding for non-commercial studies or full funding for all costs for commercial contract studies.^17^

GP practice population deprivation was measured using data originally used by NHS North of England Commissioning Support (NECS) to identify Deep End practices in NENC. This adopted the same methodology as the original Deep End project in Scotland,^10^ using quarterly GP practice lists and the Indices of Multiple Deprivation (IMD 2019 Lower-level Super Output Areas [LSOA]) to rank all GP practices by the percentage of their practice population living in the most deprived 15% of neighbourhoods (LSOAs) in England as of July 2021.^15,18^ Deep End practices are classified here as those in the decile of practices nationally with the highest proportion of patients living in these 15% most socioeconomically disadvantaged neighbourhoods. (In 2022 NENC Deep End practices had between 57.2% and 95.6% of registered patients living in the most deprived 15% of LSOAs.)

### Participant recruitment

Fifteen GPs and practice managers or administrators took part in semi-structured qualitative interviews. A sampling frame for purposive recruitment was developed based on the results of quantitative analysis. This aimed to recruit GPs and practice managers or administrators from Deep End practices with or without experience of recruiting patients to research; and from non-Deep End practices with deprivation within the practice population with experience of recruiting patients to research. Participants were recruited via email invitation either directly or cascaded via colleagues.

### Data collection

Aggregate research recruitment data for 358 GP practices in NENC were extracted from NIHR RDN ODP for financial years 2018–2019 to 2021–2022. Data earlier than 2018–2019 were not used owing to extensive prior clinical commissioning group (CCG) and practice mergers. Data for each practice were matched, accounting for practice mergers, duplicates, and missing data. Figures were combined across years to reflect overall recruitment to portfolio studies before and during the COVID-19 pandemic, and calculated as a proportion of each general practice list size using NHS England data from March 2022. The practice population deprivation percentage and national decile for each GP practice was matched based on data originally used to identify Deep End practices in NENC.

Interviews were semi-structured, facilitated using a topic guide (Appendix A), which was piloted and refined during early stages of data collection. Interviews explored participants' experience of research, and barriers and facilitators to research involvement for GP practice teams, including recruitment of patients in deprived areas. They also explored how research involvement could be supported for practices working with socioeconomically disadvantaged populations. Individual interviews lasted 20–30 minutes and were carried out via Microsoft Teams or telephone. These were conducted and field notes taken by a member of the evaluation team experienced in conducting interviews as part of applied qualitative evaluation. Interviews were audio-recorded with participants' consent, and transcribed verbatim.

### Analysis

Quantitative data were analysed to understand the relationship between GP practice deprivation and research recruitment in NENC. The data were aggregated by grouping NENC GP practices into national IMD quintiles (those within the 20% most deprived to the 20% least deprived practices in England). One-sample proportion *z*-tests were used to calculate 95% confidence intervals (CIs) for the research recruitment rate of these practice groupings. χ^2^ tests were used to investigate whether differences in the proportion of list size recruited to portfolio studies were significant at the 5% level, between quintiles of deprivation, and between Deep End and non-Deep End practices within the 20% most deprived practice grouping using Minitab (version 21.2.0).

Interview transcripts were coded by the interviewer and thematically analysed using NVivo (version 14.23.1). Codes were developed from the data 'bottom-up'; these codes were then approached deductively and categorised into themes relating to barriers and facilitators to research involvement. Themes were discussed with the wider evaluation team and steering group in the context of roundtable discussions about barriers and facilitators to research participation with NENC Deep End GP practice teams at an in-person event held in April 2023.

## Results

### Association between deprivation and research recruitment

There was an association between deprivation and recruitment to RDN portfolio studies (χ^2^(4) = 7520.8, *P*<0.001), with a graded increase in the proportion recruited per quintile of deprivation. The lowest levels of research recruitment were in practices where more patients are living in the most deprived areas (see Table 1, Figure 1), with individuals in the least deprived quintile recruited at a rate more than 12 times that of the most deprived.

**Figure 1.**
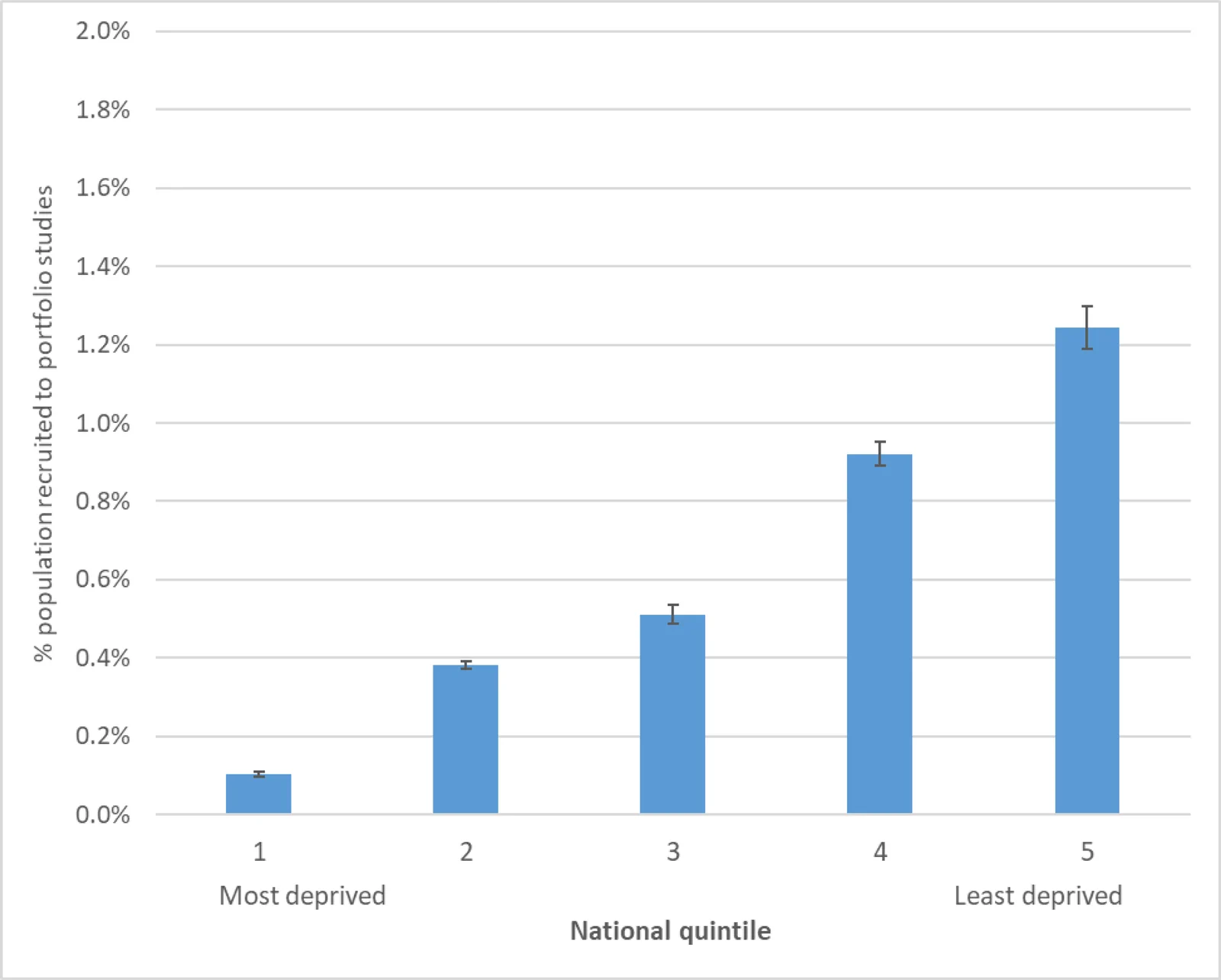
General practice recruitment to National Institute for Health and Care Research (NIHR) Research Delivery Network (RDN) portfolio studies by national Index of Multiple Deprivation (IMD) quintile of GP practices in North East and North Cumbria (NENC)

**Table 1.** Percentage of general practice population recruited to NIHR RDN portfolio studies by national IMD quintile of GP practices in NENC

National IMD quintile of GP practice	Practices	Total population	RDN portfolio recruitment 2018–2019 to 2021–2022	%	95% CI
Most deprived 1	115	982 771	1013	0.10%	0.10% to 0.11%
2	118	1 233 715	4710	0.38%	0.37% to 0.39%
3	47	362 068	1852	0.51%	0.49% to 0.54%
4	52	404 075	3723	0.92%	0.89% to 0.95%
Least deprived 5	26	166 493	2070	1.24%	1.19% to 1.30%

IMD = Index of Multiple Deprivation. NENC = North East and North Cumbria. NIHR RDN = National Institute for Health and Care Research Research Delivery Network.

Within the most deprived quintile (20% most deprived practices nationally), there was a difference in recruitment to RDN portfolio studies between Deep End (10% most deprived, *n* = 36, recruitment 0.08%, 95% CI = 0.07 to 0.09%) and other practices (*n* = 79, recruitment 0.11%, 95% CI = 0.11 to 0.12%) , compatible with lower levels of recruitment to portfolio studies in practices with the highest levels of blanket socioeconomic deprivation (χ^2^(1) = 28.3, *P*<0.001).

#### Interview participant characteristics

Eleven GPs and four practice managers or administrators were interviewed between 5 December 2022 and 21 April 2023 with individual interviews lasting 27 minutes on average. Interview participants worked in a range of geographic localities across the NENC region in practices serving communities experiencing urban, rural, and coastal deprivation, with general practice experience ranging from early to late career. Ten interview participants worked in Deep End practices; five of these participants had direct experience of RDN portfolio research being delivered in their practice either currently or in the past. Five interview participants worked in non-Deep End practices with experience of engaging more socioeconomically disadvantaged patients in NIHR portfolio research delivery within their practice. Interview participant characteristics are shown in Table 2.

**Table 2.** Interview participant characteristics

Role	Deep End	Non-Deep End
Locum GP	0	1
Salaried GP	4	0
GP partner	4	2
Practice manager or administrator	2	2
Total	10	5

There were differences in the practice populations served by the interviewees; for example, some practice populations were predominantly White British, whereas others served predominantly minority ethnic groups including people seeking asylum and refugees.

### Themes

Barriers for practice research involvement were categorised into five themes (more difficult to recruit patients; research as ‘a luxury’; risk; limited financial benefit; and limited awareness of opportunities) and supporting factors were categorised into six broad themes (relevant research; supporting patient research participation; more accurate research costing; a designated person; full remuneration; and clearer communication of ‘gives and gets’). These themes were broadly complementary, as shown in Table 3.

**Table 3.** Summary of barriers and supporting factors for research involvement in Deep End practices

Broad area	Barriers	Supporting factors
Patient need	More difficult to recruit patients Difficult patient circumstances Patient characteristics Additional resources needed to support	Relevant research Supporting patient research participation Accessible study information Additional staff time for recruitment Building trust Personalised follow-up Opportunistic approaches Providing expenses upfront
Resource	Research as ‘a luxury’ Risk	More accurate research costing A designated person
Reward	Limited financial benefit	Full remuneration
Communication	Limited awareness of opportunities	Clearer communication of ‘gives and gets’

### Barriers

#### More difficult to recruit patients

Participants highlighted barriers to socioeconomically disadvantaged populations participating in research, including lacking the ability or means to take time from work, childcare responsibilities, travel costs, and language barriers. It was acknowledged health problems and health research may not be a priority for people with difficult lives:

*‘If they’re struggling, they are single parent families, or someone is working multiple jobs to make ends meet, research might be the last thing on their mind.*’ (P05 Non-Deep End GP partner)

Additional time and resources needed to support patient recruitment, such as providing verbal information and translation services, were emphasised. In this context, lack of staff time, rather than patient willingness to participate, was seen as the main obstacle:

*‘... my guess is that that is related to how much funding and how much time the GPs have got, and less about whether patients would be happy to be recruited.*’ (P17 Deep End salaried GP)

#### Research as ‘a luxury’

Participants working in Deep End practices described complex clinical workloads, high demand for patient consultations and recruitment challenges, resulting in staff being *‘in firefighting survival mode*’, with research viewed as *‘a luxury*’ (P16 Deep End GP partner). This can make it harder to prioritise research engagement amid daily pressures:

*‘... clinicians in Deep End practices have much less time and cognitive bandwidth to lift their eyes to the horizon.*’ (P06 Deep End GP partner)

#### Risk

Participants from Deep End practices reported hesitancy to engage with research owing to previous experience and risks linked to uncertainties. These included lacking confidence in staff time requirements, whether remuneration would account for this, additional professional responsibilities, and inconsistency in research opportunities:

*‘... you could be ending up costing your practice money, which is not what you want to do and that’s detrimental potentially to patients … a lot of administration, a lot of responsibility … it’s not guaranteed enough, and there’s so many potential, practical and other problems that it didn't feel worthwhile.*’ (P17 Deep End salaried GP)

Potential for research to generate additional workload in a pressured working environment was highlighted as a particular issue:

*‘... you had to be the approved researcher to do it* [research tasks] *… which was fine when I had a bit more time but when I didn’t have time it was just a real pressure I didn’t need.*’ (P16 Deep End GP partner)

#### Limited financial benefit

Concerns were raised regarding practice remuneration for research recruitment that does not change to reflect effort involved among participants with experience of research. This led to some Deep End practices judging staff time invested in research as *‘not economically viable*’ (P16 Deep End GP partner) owing to being less likely to break even or benefit financially from research participation:

*‘I could have done the normal research in* [Non-Deep End practice] *and got the same kind of remuneration with almost 30% less work.*’ (P15 Deep End GP partner)

#### Limited awareness of opportunities

Lack of awareness of research opportunities was a barrier, with research emails often overlooked or filtered by managers to protect clinical staff time:

*‘... our practice manager, with our full backing, will just not forward things to us because she knows it’s just another thing … if you make an offer to a practice that’s financially viable, that’s the point, then that’s how you get in.*’ (P06 Deep End GP partner)

Difficulty understanding what is required of practices from research communications was also highlighted.

### Supporting factors

#### Relevant research

Participants discussed the importance of research designed to be immediately relevant to practices in socioeconomically disadvantaged areas to encourage involvement. Study designs that implicitly exclude deprived populations, through inadequate consideration of language barriers or complexity of participant information, were criticised. Research projects building on co-designed Deep End priorities were highlighted as relevant:

*‘I think what matters the most to people on the ground is having research or being involved in it that actually will have an immediate outcome on what they’re practising or experiencing.*’ (P04 Non-Deep End locum GP)

#### Supporting patient research participation

In socioeconomically disadvantaged areas where lower levels of literacy and language barriers are more common, providing accessible study information that is short and easy to read, and allocating staff time to go through information verbally or via a translator (recognising that translated conversations can take two to three times as long) was considered best practice for research recruitment. This was considered key to informed consent, particularly where patients are embarrassed to disclose reading difficulties:

*‘... even the very basic consent form ... we had to have a health care assistant going through it with the patient because they would just struggle on their own.*’ (P06, Deep End GP partner)

Building trust and understanding of health research within the NHS was described as key when working with populations new to the UK, as the word ‘research’ can carry different cultural connotations:

*‘... their vision was that they* [researchers] *would give you different drugs and use you as a guinea pig.’* (P15 Deep End GP partner)

Personalised follow-up of patients was seen as an effective research recruitment and participation strategy building on existing relationships:

*‘... they do it because they know you, as opposed to just sending out a random letter.*’ (P14, Deep End practice manager or administrator)

Integrating research recruitment and delivery within routine care was also considered key to make participation more opportunistic and less time and resource-intensive for socioeconomically disadvantaged populations:

*‘We did a joint* [research/healthy heart checks] *clinic which worked really, really well ... we wanted to have a very free flow so that the patients don’t have to then come again.*’ (P15 Deep End GP partner)

Practical support such as upfront travel expenses was also considered to be important for supporting participation.

#### More accurate research costing

Participants stressed the need for more accurate research costings to account for clinical and administrative time, potentially involving general practice teams working in areas of socioeconomic disadvantage to support development of realistic estimates:

*‘... costing properly, what it would cost the practice in terms of the clinical time if you’re asking a nurse to do something extra. But also all the admin time, interrogating records and filling in inclusion exclusion criteria ... even funding time for people being interviewed for qualitative stuff.*’ (P06 Deep End GP partner)

For practices where recruitment of staff is an ongoing challenge, sourcing staff to backfill was suggested as a means of resourcing research involvement:

*‘I mean we can’t find* [recruit] *people ... bodies on the ground would be welcome.*’ (P12 Deep End GP partner)

#### A designated person

Having a designated person with protected time to assess opportunities, coordinate, support, and deliver research within practices was widely suggested. There were varied opinions as to whether support should sit at practice level, within the NIHR RDN, or in-between. Those with experience of working with RDN research nurses shared positive experiences of working as part of a team on site:

*‘... we had* [research nurse] *associated with us, that worked brilliantly. I think if there’s a named person, practices find it much easier to communicate with them and to build that team rapport.*’ (P15 Deep End GP partner)

#### Full remuneration

Remuneration to reflect additional effort involved was deemed essential for encouraging practice research participation in socioeconomically disadvantaged areas. This was discussed primarily from a financial perspective, although remuneration ‘*in kind*’ via service provision or equipment to benefit patient care was also suggested as an option; a study involving provision of a clinical psychology service to patients was highlighted as an example.

Within the current system, rewards for participating in research were discussed to make up the shortfall, and in the context of incentivising practice participation. More research-experienced participants noted potential for commercial research to boost practice income and potentially support staff recruitment and retention challenges:

*‘... perhaps the deprived practices just need more financial support and more support to actually undertake the research ... if you could then ensure that at least some of the research they do is commercial, where they get money into the practice to do with what they wish, whether that be to attract more staff or just to top-up the salary of GPs so they remain working in that area.*’ (P02 Non-Deep End Practice GP partner)

#### Clearer communication of ‘gives and gets’

There was agreement that research communications need to be shorter, with more straightforward practical content, preferably from a trusted research contact. Key information was summarised as ‘*gives and gets*’, which is what the research offers the practice and what practices need to provide in terms of staff, sessions, and space, within a defined timeframe:

*‘... a thing that says, “We want, whatever, a session a week of your nurses’ time, or whatever, for three months and we’re going to pay you this much for it.”*’ (P06 Deep End GP partner)

## Discussion

### Summary

To our knowledge, this is the first published analysis of the relationship between research participation and neighbourhood deprivation at scale; made possible by assessing the relationship between general practice population neighbourhood deprivation data and research recruitment. Analysis shows research recruitment is lower among practices with more deprived populations, and significantly lower among practices working in areas of blanket deprivation (*P*<0.001). Qualitative findings suggest research is *‘a luxury*’ in Deep End practices owing to high volume of complex patient need and limited resources. Socioeconomically disadvantaged patients are perceived as willing potential research participants, but additional time and resources are needed to support research participation, meaning more effort is required to recruit to research in these practices.

### Comparison with existing literature

The finding that primary care research recruitment is lower in GP practices with more deprived populations is consistent with evidence that diseases common in socioeconomically disadvantaged communities are disproportionately studied in healthier populations.^4^

This evaluation highlights potential disadvantages of practice research participation in areas of blanket deprivation, including time challenges, which reduce GP ability to manage their usual clinical workload. This differs from a recent study exploring general practice research activity, which found few disbenefits of general practice research participation.^19^ It is aligned with challenges reported by Deep End practices that include a high burden of ill health and patient need, difficulties with staff recruitment and time for professional development.^20–22^

These challenges may exacerbate common barriers to general practice research participation identified in existing literature, including lack of time, administrative burden, poor research communications, and concerns about possible patient harms.^23,24^ Findings are also in agreement with recent studies that identify support from local research networks, funding incentives,^19,25^ and involvement in research development^23^ as supporting factors for general practice research delivery.

This evaluation identifies additional issues in deprived areas: limited research opportunities that are adequately costed, resourced, and designed to meet the needs of socioeconomically disadvantaged populations. Although there is limited literature that directly captures patient experience of primary care research in socioeconomically disadvantaged areas, findings are consistent with known barriers to research participation in this population, including not understanding research information, difficulty with transport, and having other priorities of daily living.^26^ It is argued written invitations to participate have low conversion rates among socioeconomically disadvantaged communities.^9^ Low health literacy,^27^ expensive public transport, and lack of time^19^ are also identified barriers to socioeconomically disadvantaged populations accessing health care. This study provides relational insights as to how general practice can support patient research participation in areas of deprivation, complementing existing evidence on aspects of research design.^26,28–30^

Taking a wider health equity lens, the findings of this evaluation are aligned with theory that recognises the interplay between system-, service-, and patient-level factors as determining inequities in health and health research.^12^ Findings reflect the link between service-level factors (such as management processes that limit awareness of research opportunities) and health service and research structures that do not sufficiently resource research participation for general practices in socioeconomically disadvantaged areas. Patient-level factors extended beyond perceptions of individual preferences (such as deprioritising health amid life stressors), reflecting broader societal inequalities (including employment conditions, caregiving, travel costs, and literacy levels), which shape an individual’s ability to engage with health care and research. Findings highlight the approach to research communication within clinical encounters as key to improving inclusivity. In the context of recent literature on reducing health inequalities, factors amenable to change include development of health research policy structures, which support equitable participation and inclusive and flexible approaches to research in socioeconomically disadvantaged areas in consultation with practice and patient communities.^13,14^

### Strengths and limitations

Data were matched and analysed for all practices in NENC, providing a large sample size and opportunities for subgroup analysis. Analysis was structured using national rankings of practice-level deprivation, which allows for some contextualisation, but assessment of applicability to other contexts should consider population demographics and need,^31^ socioeconomic deprivation, and levels of primary care research recruitment. Univariate analysis was used to understand the association between practice deprivation and research recruitment. Further covariate analysis was beyond the scope of this evaluation; however, it could assess the impact of other factors on recruitment such as practice size, population age, ethnicity, and proximity to hospitals with a strong research culture. Recruitment to NIHR RDN portfolio studies was used as a measure of research participation; this excludes projects where funding or activity does not meet NIHR eligibility criteria such as audit, service evaluation, and aspects of academic practitioner roles.^17^ Research recruitment figures were combined over several years; this did not distinguish pre- and post-COVID recruitment, and was still a small proportion of the registered population.

The quantitative analysis was used to inform the qualitative evaluation sampling frame. Recruitment included participants with a range of practice and research experience, which was considered important to understand how research could be developed in future. This was supported by including notes from discussions at a well-attended event. Although comprehensive mailing lists were used to contact potential participants, qualitative recruitment was most successful where there was a mutual connection with the evaluation team; the sample may reflect those most likely to engage with evaluation and research opportunities. While there was common repetition of barriers and supporting factors across interviews, this was not as strong among participants with more experience of research.

The evaluation did not include patient perspectives. Findings in relation to patient research involvement are based on perceptions of general practice staff with experience of working with patients in deprived areas, who are not always able to judge the level of socioeconomic disadvantage individuals are experiencing at the point of care.

### Implications for research and practice

For the benefits of primary care research participation to be distributed more equitably, research should be accurately costed and remunerated to reflect effort involved for practices working with socioeconomically disadvantaged populations; involve an additional service or human resource; and be designed to meet needs of socioeconomically disadvantaged patients. Further research to inform practice is recommended, including multivariate analysis to enable a more nuanced understanding of the relationship between socioeconomic deprivation and health research recruitment and qualitative research to capture patient experience of research participation in socioeconomically disadvantaged areas.

Current regional initiatives include a dedicated research nurse, employed by NIHR NENC Regional RDN to provide additional focused support for research engagement and participation among practices and patients in areas of blanket socioeconomic deprivation. The NENC DE Network prioritises applied research to generate evidence about what works and develop research-practice partnerships.^22,32,33^ More widely, Deep End Network Fellowship opportunities are designed to support newly qualified GPs to engage in projects aimed at reducing health inequalities.^33,34^

Support to increase general practice research engagement in the most socioeconomically disadvantaged areas is more likely to be effective in the context of wider infrastructure that promotes and incentivises equitable and consistent general practice research opportunities. The NHS England Research Engagement Network initiative offers integrated care boards the opportunity to develop and implement programmes to support research participation among underserved communities. In the medium to long term, research policy and enforcement could be effective levers for change,^6,7^ particularly strategic investment, pre-funding assessment processes, and monitoring of outcomes. Recent NIHR priorities include establishing inclusion as a condition for health research funding and implementing equity monitoring across funded studies.^35^ Further assessment of how best to utilise national research infrastructure to ensure equitable primary care research and minimise unintended consequences is recommended.

## Data

The dataset relied on in this article is available from the corresponding author on reasonable request.
